# A Quantitative Framework for Measuring Personalized Medicine Integration into US Healthcare Delivery Organizations

**DOI:** 10.3390/jpm11030196

**Published:** 2021-03-12

**Authors:** Arushi Agarwal, Daryl Pritchard, Laura Gullett, Kristen Garner Amanti, Gary Gustavsen

**Affiliations:** 1 Health Advances LLC, San Francisco, CA 94105, USA; 2 Personalized Medicine Coalition, Washington, DC 20036, USA; dpritchard@personalizedmedicinecoalition.org; 3 Health Advances LLC, Newton, MA 04266, USA; lgullett@healthadvances.com (L.G.); kgamanti@healthadvances.com (K.G.A.); ggustavsen@healthadvances.com (G.G.)

**Keywords:** precision medicine, personalized medicine, maturity model, health system, healthcare delivery

## Abstract

Personalized medicine (PM) approaches have revolutionized healthcare delivery by offering new insights that enable healthcare providers to select the optimal treatment approach for their patients. However, despite the consensus that these approaches have significant value, implementation across the US is highly variable. In order to address barriers to widespread PM adoption, a comprehensive and methodical approach to assessing the current level of PM integration within a given organization and the broader healthcare system is needed. A quantitative framework encompassing a multifactorial approach to assessing PM adoption has been developed and used to generate a rating of PM integration in 153 organizations across the US. The results suggest significant heterogeneity in adoption levels but also some consistent themes in what defines a high-performing organization, including the sophistication of data collected, data sharing practices, and the level of internal funding committed to supporting PM initiatives. A longitudinal approach to data collection will be valuable to track continued progress and adapt to new challenges and barriers to PM adoption as they arise.

## 1. Introduction

Over the past decade, scientific and technological advances have vastly expanded the tools and treatments available to physicians for screening, diagnosing, treating, and monitoring patients based on their individual circumstances and molecular characteristics. A better understanding of the molecular drivers of disease, coupled with the rapidly improving logistics and economics of genetic and genomic testing, has elevated the quality of care provided in a number of clinical areas. These approaches can improve clinical outcomes and reduce costs [1]. Yet, while research and innovation in personalized medicine (PM), also commonly referred to as “precision medicine,” is surging, its adoption into clinical practice has been comparatively slow.

Recognizing its value in clinical management, providers are increasingly working to integrate PM into their health care work streams. Led by several pioneering healthcare institutions, health systems, and independent hospitals and clinics across the United States are adopting strategies and processes to implement PM approaches into clinical care. These efforts are most advanced in oncology but are also gaining traction in other areas, such as for the diagnosis of rare diseases and for informing treatment decisions in some chronic conditions. Ultimately, researchers hope PM will guide the proactive screening of healthy patients and inform treatment strategies based on a wider set of biological and environmental data points. Pilot studies to validate the utility of these approaches are ongoing [2,3].

Despite these efforts, it is not clear what impact the move toward personalized medicine has had so far on the health care system in general. Novel challenges associated with the evolving field of PM have led to a lag in the adoption of the approach at many healthcare delivery organizations. Key barriers to the successful integration of PM have historically included education, informatics, patient engagement, internal funding, and ensuring high-value testing and data collection practices [3]. The field’s leaders have made considerable progress in addressing some of these barriers, but many health care delivery institutions have significant work remaining to ensure that patients benefit from the clinical and economic advantages of PM to the fullest extent possible [4,5,6].

Assessing the current landscape of PM integration in healthcare delivery organizations can help guide clinical adoption efforts by providing technology developers and policymakers with a holistic view of practice patterns and barriers related to the utilization of PM and by defining a standard that individual healthcare delivery institutions may use to benchmark their own efforts. Conducting such an assessment requires a framework useful for evaluating the state of PM integration across organizations. Such a framework must take into account the variation among organizations in terms of the clinical areas in which PM is employed, as well as the testing, informatics, and leadership that enable these efforts. When applied to a representative sample of US healthcare organizations, this framework will enable the longitudinal evaluation of PM integration efforts across the country and help identify the continued impact of known barriers (e.g., informatics and internal funding) as well as emerging needs. This, in turn, will inform efforts to address the most critical challenges to widespread implementation.

This report describes the development of such a framework and its application to a diverse sample of 153 healthcare organizations (health systems, independent hospitals, and integrated delivery networks) to gauge the level of PM integration across the US.

## 2. Materials and Methods

We conducted a survey of health care delivery institutions, specifically including health systems, independent hospitals, and integrated delivery networks, to inform the calculations of a quantitative framework that assesses progress toward PM integration at the institutional level. To guide and inform the development of the framework and survey instrument, we conducted in-depth interviews with key stakeholders at four healthcare organizations in the US. These organizations included two community health systems, one integrated delivery network, and one academic health system; because these interviews were conducted on a double-blinded basis, we are unable to reveal the names of the institutions that were interviewed. All of these organizations are widely recognized as leaders in PM, and each can be credited with spearheading novel initiatives to drive adoption within their institutions. The primary objectives of these interviews were to gain a deeper understanding of how the systems have adopted PM, understand the challenges associated with the clinical integration of PM, and develop a perspective on the range of PM adoption across these different institutions.

These interviews served as a foundation to identify what parameters were valued by institutions at the leading edge of PM and the key metrics by which these organizations were measuring the level of PM adoption. Based on these interviews, we defined eight evaluation criteria for the framework. Three of these criteria reflect the testing performed and data collected to enable personalized medicine. The remaining categories were testing guidance and data accessibility, leadership support, internal funding, utilization of data, and data sharing efforts.

To account for the differential progress that underpins PM in various clinical areas, we determined that each healthcare organization would be surveyed against the chosen criteria in five clinical areas: oncology, prenatal/neonatal screening, pharmacogenomics/chronic disease, rare/undiagnosed disease, and healthy patient screening. Composite evaluations combined our survey measures of the extent of integration in each of these clinical areas.

**Figure 1:**
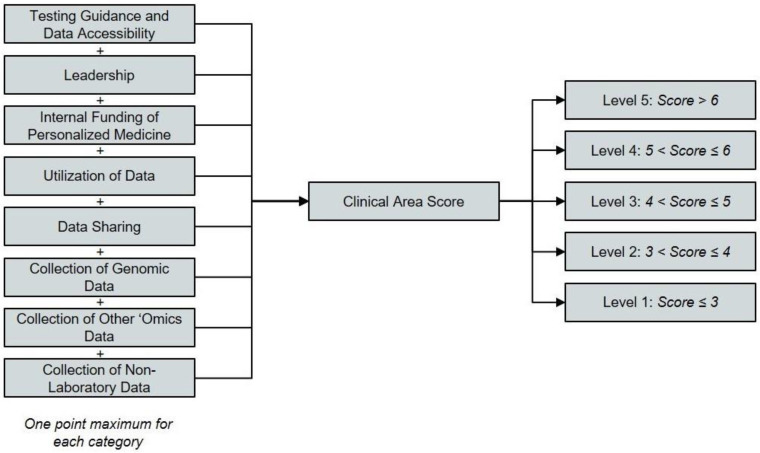
Assignment of the level of personalized medicine integration for one clinical area.

The complete framework that was applied to assess each institution’s progress in each of the five clinical areas is presented and described in Table 1 and Figure 1. A point system was used to show the extent of PM integration at survey respondent institutions. Up to one point was attributed to the health system for each criterion. Three different approaches are used to assign points across the eight criteria. For three criteria, a score of 1/3 of a point, 2/3 of a point, or one full point are assigned based on the discrete category that best describes a health system (Table 1, Rows 1–3). The criteria subject to this first scoring technique are testing guidance and data accessibility, leadership, and funding of personalized medicine. For two additional criteria, specifically utilization of data and data sharing, 1/3 or 1/4 of a point, respectively, are assigned for satisfying each of the qualifying statements assessed for the criterion in question (Table 1, Rows 4–5). Organizations meeting all three of the qualifications for data utilization or all four of the qualifications for data sharing can score a full point.

The remaining criteria capture the types of testing performed and data collected to enable PM. These criteria are scored by a two-step process. First, a baseline score of 1/9 of a point, 2/9 of a point, or 1/3 of a point is assigned for each of these criteria based on the most advanced data collection and analysis technique employed for each data type (Table 1, Rows 6–8). This baseline score is then multiplied by a factor of one, two, or three to account for the consistency of that data collection (Table 1, Row 9). The consistency of data collection is gauged based on whether some, most, or all physicians collect that type of data for their patients. For example, an organization that collects genomic data (Criterion No. 6) using multigene hotspot panels (the most sophisticated technique employed by the institution in question) will earn a baseline score of 2/9. If those data are ordered by most physicians (a multiplicative factor of two), the institution would receive a final score for this criterion of 4/9. If those data are ordered by all physicians (a multiplicative factor of three), the institution would receive a final score for this criterion of 6/9 (Table 1).

The point totals for all eight criteria in a given clinical area are then summed and categorized according to the rubric of five levels outlined in Figure 1. Institutions scoring three or less in any given clinical area are considered “Level 1” implementers in that clinical area (the lowest possible level), while those scoring more than six are considered “Level 5” implementers (the highest possible level). Organizations that are considered “Level 0 self-reported that they do not implement any PM approaches into care for any clinical area.

**Figure 2:**
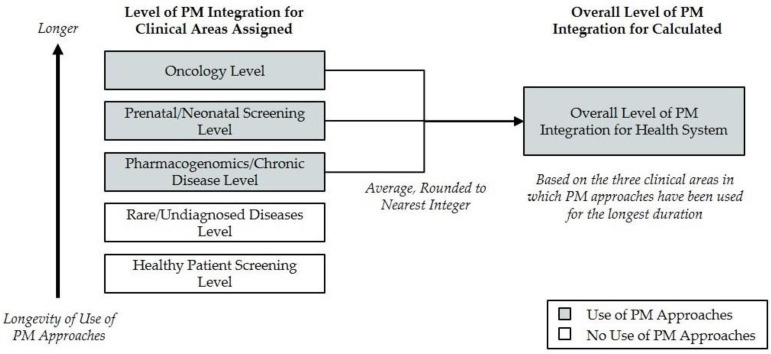
Example assessment of the overall level of personalized medicine integration for a health system.

The overall level of PM integration across the organization is defined as the average of the levels calculated for the three clinical areas in which the health system has been employing PM approaches for the longest duration, rounded to the nearest integer (Figure 2). If a health system is employing PM approaches in only one or two clinical areas, the health system would be assigned “Level 0” for two or one clinical areas, respectively, in order to calculate the average. This approach intentionally penalizes systems that have not integrated personalized medicine approaches broadly among clinical areas. Although different stakeholders may have a greater interest in particular areas of healthcare delivery, the diverse aspects considered in this framework are all believed to be critically important to understanding the overall level of PM integration, and as such, each criterion is weighted equally in this analysis.

As indicated above, we applied this framework to assess the level of PM integration across the United States using data collected from an online survey of stakeholders involved in PM initiatives at US health systems. To ensure high market research quality, the 153 survey respondents were recruited by a market research vendor in compliance with ISO 26362 International Standards. Survey respondents represented a variety of health system types and roles, including lab directors, CEOs, Chief Medical Officers, and Chief Information Officers (Table 2). All respondents were actively involved in PM initiatives. Over the course of the survey, respondents answered questions that enabled scoring against each criterion in the framework for their health system for up to three clinical areas (Questionnaire S1). The survey data were then analyzed to generate a quantitative picture of PM integration in the US.

## 3. Results

**Figure 3:**
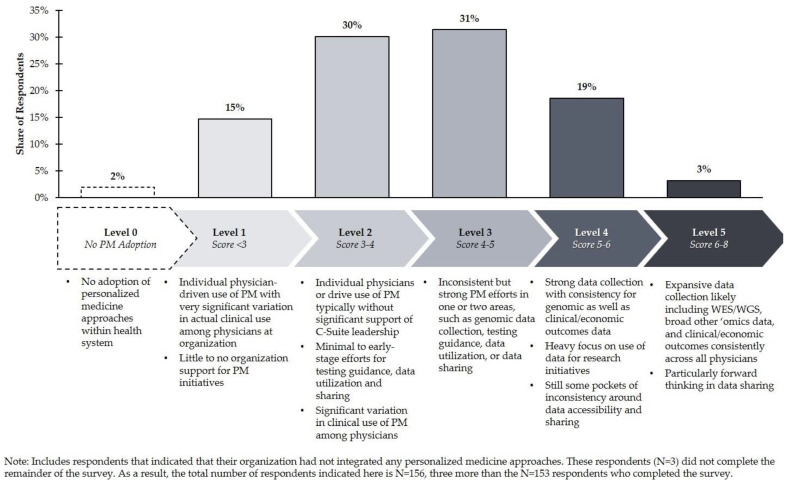
US health system distribution of the overall level of personalized medicine integration.

**Figure 4:**
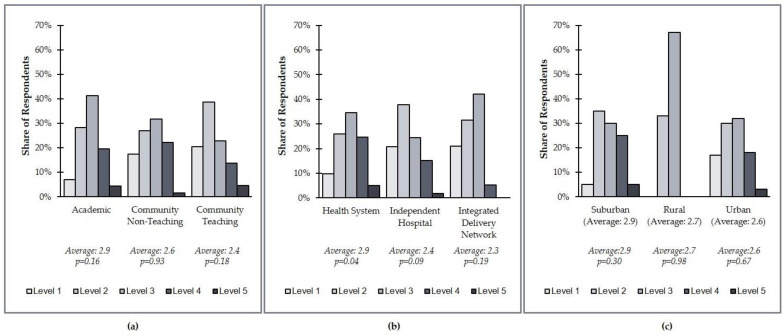
US health system distribution of the overall level of personalized medicine integration by affiliation, practice type, and practice demographics. (**a**) Overall level of personalized medicine integration by affiliation; (**b**) overall level of personalized medicine integration by practice type; (**c**) overall level of personalized medicine integration by practice demographics.

This research found that although US healthcare organizations are widely distributed in terms of their composite level of PM integration, most are at Level 2 or Level 3 (Figure 3). Academic organizations are less likely to be at Level 1 compared to other health system types. In fact, only 7% of academic organizations are at Level 1, while 17% of community nonteaching and 20% of community teaching organizations are at Level 1. By organization type, integrated delivery networks (IDNs) were less likely to be at a Level 4 or 5 than health systems or independent hospitals. Only 5% of IDNs were ranked at Level 4/5, vs. 30% of health systems and 17% of independent hospitals (Figure 4). It is important to note, however, that this result is based on a limited sample size of IDNs (*N* = 19 total survey respondents).

**Figure 5:**
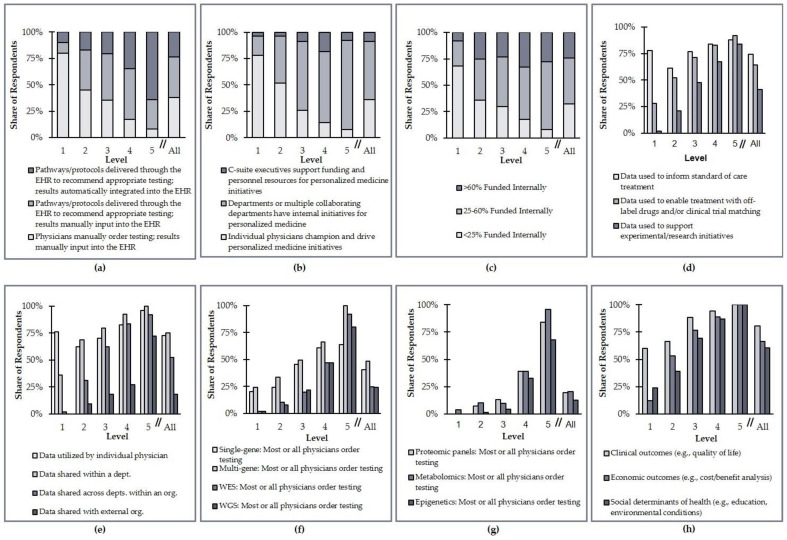
US health system distribution of the overall level of personalized medicine integration by criteria. (**a**) Testing guidance and data accessibility; (**b**) leadership; (**c**) internal funding of personalized medicine; (**d**) utilization of data; (**e**) data sharing; (**f**) collection of genomic data; (**g**) collection of other omics data; (**h**) collection of non-laboratory data.

The 153 respondents to this survey indicated a total of 433 PM programs in various clinical areas within their organizations. Each of these programs was assigned a level of PM integration that contributed to the organization’s composite level, as described in the Methods section (Section 2). The data for these clinical area-specific programs are displayed in Figure 5. These data indicate significant heterogeneity in how organizations have advanced PM integration efforts at their institutions. For example, 24% of Level 1 organizations collect data on social determinants of health to aid their PM efforts, while only 8% of Level 5 organizations manually order testing and manually input results into the EHR.

Most of the criteria analyzed do not differ substantially when split by clinical area. The collection of genomic data and the utilization of those data, however, are more common in some clinical areas than others. Specifically, whole-exome sequencing (WES) and whole-genome sequencing (WGS) are most commonly ordered for the diagnosis and treatment of oncology and rare/undiagnosed diseases (Table 3). In terms of the utilization of data, more established clinical areas such as oncology and prenatal testing also tend to focus more on collecting actionable data; other clinical areas are more focused on research and experimentation (Table 4).

A correlation analysis between the clinical area criteria scores and the level classification was performed to assess the impact that certain criteria had on indicated levels. Correlation coefficients were generally greatest for criteria related to the collection of non-laboratory data, data sharing, and the collection of other omics data across clinical areas, indicating that these variables were more highly interdependent with the level classification. Correlation coefficients for leadership, testing guidance, data accessibility, and funding were, in general, less interdependent with level classification (Table 5).

## 4. Discussion

This research establishes a first-of-its-kind framework to assess the landscape of PM integration within the US healthcare system at an institutional level [7]. While consensus is building that adoption is continuing to expand, the evidence for these claims remains largely qualitative. Quantitative studies have primarily focused on assessing the use of biomarker testing for targeted therapy selection in oncology [8,9,10]. This broader framework can therefore help inform providers and technology developers’ PM implementation efforts and can serve as a resource for health care delivery institutions to examine best practices for PM, evaluate internal organizational programs and practices, and identify areas of focus to address outstanding integration challenges. This analysis is not limited to the evaluation of a single clinical area but instead recognizes and informs all key clinical areas in which PM is rapidly advancing.

This framework enables a quantitative evaluation of PM integration efforts across different healthcare delivery institution types, sizes, communities served, and/or geographical regions. The framework can be applied to illustrate the structure and status of PM at various health systems and highlight how well systems may be addressing key challenges to PM integration.

The landscape analysis applied the framework to a representative sample of 153 organizations across the United States to develop a quantitative picture of PM integration across the country. Repeating this study under similar conditions in the future would enable longitudinal analyses of how PM integration is advancing across the US. Such a longitudinal analysis would also serve to identify areas in which PM implementation efforts are having the greatest impact and, conversely, the areas in which additional investment is required.

This analysis indicates that PM approaches are widely used among US healthcare organizations today. This marks a significant shift within health care from traditional one-size-fits-all medicine to the delivery of personalized care that can take into account individual patient characteristics to provide more effective and efficient tailored treatment strategies. In many cases, PM data have enabled organizations to look beyond the standard of care and explore off-label, trial, or other research alternatives to optimize care for patients. The vast majority of health care delivery institutions are collecting various levels of individual molecular information but also at least one type of non-laboratory data to enable PM, such as those on social determinants of health.

This study also suggests, however, that PM approaches are often only used at a basic level and are not actively supported by the organization. PM integration often only penetrates certain departments within an institution. Top-down leadership is uncommon. The 15% of organizations that were classified overall as Level 1 lacked system-wide advanced diagnostic testing guidance and typically incorporated PM efforts that were largely driven by individual physician leaders. Adoption of PM strategies within these institutions typically varied significantly depending on individual clinician practice patterns, as made evident through a survey of lab directors who are able to observe the day-to-day operations across the health care delivery institution.

Health systems currently face many challenges in integrating PM into clinical settings, the most significant of which are testing guidance, data sharing, and programmatic funding.

Challenges in organizational testing guidance, which stem from the infrastructure for data collection, exchange, and access, especially related to leveraging the EHR to effectively guide, capture, and mine data collected for PM, are an ongoing challenge [11,12]. Although most organizations have some level of EHR integration, full integration of testing recommendations and results into the EHR remains rare and a key obstacle. With the nearly ubiquitous incorporation of EHRs into daily health system activities, and the increasing engagement of their use by patients for care tracking, challenges to their use for PM may come as a surprise to some stakeholders. However, this may also represent a key area for which progress can be made quickly.

Data sharing is an area where organizations nationally have room to advance their efforts. Across clinical areas, respondents indicated that PM data collected at their organization is primarily shared internally. Relatively few organizations—only 18%—share data externally.

Access to external funding remains a consistent challenge across institutions. A total of 68% of surveyed institutions provide internal funding for at least 25% of the personalized care practiced within the institution. Internal institutional funding of PM highlights the commitment that organizations are making to implementation efforts but also points to the insufficiency of current coverage and reimbursement policies in covering the costs for personalized care.

From a testing standpoint, few organizations (less than 25%) are ordering tests to support PM that go beyond the genetic testing commonly associated with the field. The collection of non-laboratory data is more frequent, particularly in the context of the clinical and economic outcomes that are becoming an increasing interest of healthcare organizations.

Significantly, this analysis illustrates a substantial heterogeneity in the approach and structure of personalized medicine integration throughout the health care system. There is no singular organizational archetype at any given level. For example, while 72% of Level 5 institutions share data externally, 28% do not. Similarly, many physicians at some Level 1 and Level 2 institutions order whole-exome or whole-genome sequencing for their patients. Seemingly slow integration of PM throughout the broader health care system may, in part, be related to this heterogeneity of institutional implementation. Each health care delivery institution is different and may experience different magnitudes of implementation challenges. Thus, no set roadmap will be able to get every institution to its PM implementation destination.

Yet, despite this heterogeneity, the research signals some consistent factors indicative of success at higher-level institutions. Those organizations at a Level 4 or 5 pursue more sophisticated data collection, including other omics data and non-laboratory data, and proactively share this data internally and externally. Furthermore, these organizations recognize that reimbursement and other external funding sources can be challenging to secure and have taken on the responsibility of committing internal resources to support a significant portion of PM initiatives.

The findings of this research have potential limitations. While the majority of respondents were laboratory directors, several different institutional leadership roles were represented, and thus different perspectives may have translated differently to survey responses. The survey included 153 respondents from institutions of various types (health systems, independent hospitals, and integrated delivery networks) and demographics (urban, suburban, and rural), but not all types and demographics were represented equally. The number of respondents from integrated delivery networks and rural systems in particular were notably smaller than the other groups, and thus the average distributions reported are subject to lower statistical significance (*p* = 0.19).

All data collected as responses to this survey were self-reported without any follow-up verification or confirmation by the researchers. Thus, the results are subject to potential bias, as survey respondents may have over-represented PM efforts within their institutions. The group of respondents that completed this survey may also be skewed toward health system leaders and/or lab directors with a strong interest in personalized medicine and/or who work at institutions with a more systemic interest in personalized medicine integration. Conversely, potential respondents with a lower interest in personalized medicine may have opted not to complete the survey. Furthermore, those who specifically indicated their organizations did not have any ongoing PM initiatives were excluded from the survey, which may have contributed to some overinflation of the clinical adoption numbers.

## 5. Conclusions

This landscape analysis captures a holistic picture of the current state of the clinical adoption of PM strategies and technologies within the US health care system. The framework put forth in this research will become increasingly valuable as it is used repeatedly to assess the advancement of PM integration longitudinally over time. A better understanding of the evolving landscape for implementation will help clarify the extent to which PM penetrates health care practices. This, in turn, will inform efforts to address the most critical outstanding integration challenges faced by PM technology developers, providers, payers, patients, and policymakers.

## Figures and Tables

**Table 1:** Framework to evaluate personalized medicine integration for each of the five clinical areas at every institution studied.

**Criteria with Discrete Scores**	**Score**		
	**1/3**	**2/3**	**1**
1. Testing Guidance and Data Accessibility	Individual physician-driven genomic testing with manual (e.g., PDF) test ordering and results reporting	Recommended/reflexive testing pathways through the HER Manual EHR entry for test results	Recommended/reflexive testing pathways through the HER Automatic results integration into the EHR
2. Leadership	Individual physician champions drive personalized medicine initiatives	Internally focused, department-level initiatives for personalized medicine	C-Suite champions support funding and personnel resources toward personalized medicine initiatives
3. Internal Funding of Personalized Medicine	<25% funded internally	25–60% funded internally	>60% funded internally
**Criteria with Scores Based on Breadth**	**Score**		
4. Utilization of Data	One-third point to account for each of the following: Data utilization to inform the standard of care treatment Data utilization to enable treatment with off-label drugs and clinical trial matching Data utilization to support experimental/research initiatives		
5. Data Sharing	One-fourth point to account for each of the following: Data only utilized by individual physicians Data shared with a multidisciplinary team within a department Data shared across departments within an organization Data shared with external organizations		
**Criteria with Scores Based on Breadth and Consistency**	**Score**		
	For Categories 6–8, the total score will be based on the baseline score for the most advanced level of data collected. This baseline score will then be multiplied by the multiplicative factor to account for the consistency of data collection.		
	**1/9**	**2/9**	**1/3**
6. Collection of Genomic Data	Genomic data collected from disparate biomarkers	Genomic data collected from multigene hotspot panels	Genomic data collected from whole-genome or whole-exome sequencing
7. Collection of Other Omics Data	Data collected from any one of the following: proteomic, epigenetic, metabolomic	Data collected from any two of the following: proteomic, epigenetic, metabolomic	Data collected from proteomic, epigenetic, metabolomic testing
8. Collection of Non-Laboratory Data	Data collected from any one of the following: social determinants of health, clinical outcomes, economic outcomes	Data collected from any two of the following: social determinants of health, clinical outcomes, economic outcomes	Data collected from social determinants of health, clinical outcomes, economic outcomes
**Multiplicative Factor**	**1**	**2**	**3**
Consistency of Data Collection	Some physicians order for their patients	Most physicians order for their patients	All physicians order for their patients

**Table 2:** Survey respondent demographics.

Category	Share of Respondents
**Involvement in PM Initiatives**	
Spearhead/Chair PM Initiatives	41%
Member of PM Committee	52%
Well Aware of Organization’s PM Initiatives	7%
**Role**	
Lab Director	75%
CIO or CMIO	14%
CEO, CMO, or COO	11%
**Practice Type**	
Health System	53%
Independent Hospital	34%
Integrated Delivery Network	13%
**Affiliation**	
Academic	30%
Community Teaching	29%
Community Non-Teaching	41%
**Number of Hospitals**	
1	35%
2–5	34%
6–10	20%
11–25	7%
26+	3%
**Region**	
South	32%
Northeast	28%
Midwest	24%
West	16%
**Practice Demographic ^1^**	
Rural	2%
Suburban	13%
Urban	85%

^1^ Demographic classification based on the location of the facility of each respondent, corresponding to the CDC Urban–Rural Classification Scheme for Counties. Urban settings are those defined as Large Metropolitan and Medium Metropolitan (population +250 K), Suburban settings are those defined as Small Metropolitan and Micropolitan (population 10 K–249,999 K) and Rural settings are those defined as Noncore (population <10 K).

**Table 3:** Collection of genomic data.

Criteria	Share of Respondents				
	Oncology	Prenatal/Neonatal Screening	Pharmacogeno-mics/Chronic Disease	Rare/Undiagnosed Disease	Healthy Patient Screening
**Single-Gene**					
All physicians order testing	17%	11%	8%	9%	14%
Most physicians order testing	34%	19%	26%	25%	29%
Some physicians order testing	29%	35%	38%	38%	29%
No physicians order testing	19%	34%	28%	28%	29%
**Multigene**					
All physicians order testing	19%	8%	13%	11%	12%
Most physicians order testing	41%	24%	28%	35%	38%
Some physicians order testing	31%	39%	36%	35%	30%
No physicians order testing	8%	29%	23%	18%	20%
**WES**					
All physicians order testing	5%	8%	8%	14%	8%
Most physicians order testing	20%	10%	15%	20%	17%
Some physicians order testing	23%	21%	19%	32%	14%
No physicians order testing	52%	61%	58%	34%	62%
**WGS**					
All physicians order testing	6%	6%	6%	12%	3%
Most physicians order testing	22%	10%	18%	20%	14%
Some physicians order testing	29%	18%	10%	29%	21%
No physicians order testing	44%	66%	66%	28%	62%

**Table 4:** Utilization of data.

Criteria	Share of Respondents				
	Oncology	Prenatal/Neonatal Screening	Pharmacogeno-mics/Chronic Disease	Rare/Undiagnosed Disease	Healthy Patient Screening
Data used to inform the standard of care treatment	81%	82%	70%	66%	33%
Data used to enable treatment with off-label drugs and/or clinical trial matching	70%	47%	66%	71%	44%
Data used to support experimental/research initiatives	51%	21%	38%	48%	23%

**Table 5:** Correlation between criteria score and indication level.

Criteria	Share of Respondents				
	Oncology	Prenatal/Neonatal Screening	Pharmacogenomics/Chronic Disease	Rare/Undiagnosed Disease	Healthy Patient Screening
Testing Guidance and Data Accessibility	0.42	0.40	0.34	0.37	0.39
Leadership	0.44	0.47	0.34	0.39	0.33
Internal Funding of Personalized Medicine	0.30	0.22	0.20	0.29	0.35
Utilization of Data	0.51	0.58	0.59	0.44	0.58
Data Sharing	0.58	0.65	0.57	0.64	0.59
Collection of Genomic Data	0.54	0.52	0.54	0.51	0.57
Collection of Other Omics Data	0.59	0.61	0.56	0.74	0.43
Collection of Non-Laboratory Data	0.57	0.63	0.69	0.63	0.63

## Supplementary Materials

The following are available online at https://www.mdpi.com/2075-4426/11/3/196/s1, Questionnaire S1: Screener Questions for Survey Participants.
