# Supplementary material for: A Quantitative Framework for Measuring Personalized Medicine Integration into US Healthcare Delivery Organizations
Source: J Pers Med. 2021 Mar 12;11(3):196. doi: 10.3390/jpm11030196 (PMC8000405; doi:10.3390/jpm11030196)
Supplement: Supplementary file 1 [file jpm-11-00196-s001.pdf]

A Quantitative Framework for Measuring Personalized Medicine Integration into US Healthcare Delivery Organizations

Arushi Agarwal, Daryl Pritchard, Laura Gullett, Kristen Garner Amanti, and Gary Gustavsen

---

Supplementary Materials

Questionnaire S1: Screener Questions for Survey Participants

1. Which of the following best describes your primary role/department?
    a. Laboratory
    b. Biomedical/Clinical Engineering/EBME (Electro-Biomedical Engineering) **[THANK AND TERMINATE]**
    c. C-Suite Management
    d. Facilities/Maintenance/Estates Management/Building Services **[THANK AND TERMINATE]**
    e. Imaging **[THANK AND TERMINATE]**
    f. Information Technology/Informatics/Medical Informatics
    g. Nursing **[THANK AND TERMINATE]**
    h. Patient Safety/Risk Management **[THANK AND TERMINATE]**
    i. Pharmacy **[THANK AND TERMINATE]**
    j. Purchasing/Materials Management **[THANK AND TERMINATE]**
    k. Respiratory Therapy **[THANK AND TERMINATE]**
    l. Surgery **[THANK AND TERMINATE]**
    m. Clinical/Quality Research **[THANK AND TERMINATE]**
    n. Other **[THANK AND TERMINATE]**

2. Which of the following best describes your organization?
    a. Health System
    b. Integrated Delivery Network (i.e. health system providing insurance plan)
    c. Independent Hospital
    d. Government-Affiliated Hospital (e.g., VA)
    e. **[If (A) Selected in Q1]** Independent Laboratory **[THANK AND TERMINATE]**
    f. Private Practice **[THANK AND TERMINATE]**
    g. Other **[THANK AND TERMINATE]**

3. How long have you worked at your current organization?
    a. Less than 1 year **[THANK AND TERMINATE]**
    b. 1-5 years
    c. 6-10 years
    d. 16-25 years
    e. More than 25 years

4. How many hospitals does your organization have?

a. None **[THANK AND TERMINATE]**
b. 1
c. 2-5
d. 6-10
e. 11-25
f. 26+

5. Where is the headquarters of your organization?
    a. Northeast (CT, MA, ME, NH, NJ, NY, PA, RI, VT)
    b. South (AL, AR, DC, DE, FL, GA, KY, LA, MD, MI, NC, OK, SC, TN, TX, VA, WV)
    c. Midwest (IA, IL, IN, KS, MI, MN, MO, NE, ND, OH, SD, WI)
    d. West (AK, AZ, CA, CO, HI, ID, MT, NM, NV, OR, UT, WA, WY)
    e. Outside of the US **[THANK AND TERMINATE]**

6. Which of the following does your organization include? Select all that apply.
    a. Teaching Hospital(s)
    b. Clinical Trials
    c. Medical School

7. For which of the following clinical areas does your organization treat patients? Select all that apply.
    a. Oncology
    b. Rare/Undiagnosed Diseases
    c. Chronic Diseases
    d. Preventive/Healthy Patient Care
    e. Prenatal/Neonatal Care
    f. I don't know **[THANK AND TERMINATE]**

8. For which of the following clinical areas do physicians at your organization use any personalized medicine approaches? Select all that apply.

    Personalized medicine encompasses any therapeutic interventions that are tailored based on the unique physiological or biological characteristics (often genetic/molecular) of an individual or disease state.

    a. Oncology
    b. Rare/Undiagnosed Diseases
    c. Pharmacogenomics/Chronic Diseases
    d. Preventive/Healthy Patient Care
    e. Prenatal/Neonatal Care
    f. None **[THANK AND TERMINATE]**
    g. I don't know **[THANK AND TERMINATE]**

9. **[If (A) Selected in Q1]** How would you classify your primary laboratory?
    a. Research laboratory **[THANK AND TERMINATE]**
    b. Clinical laboratory

c. Other **[THANK AND TERMINATE]**

10. **[If (A) Selected in Q1]** Which of the following most closely matches your title or role?
    a. Laboratory director
    b. Laboratory manager/supervisor
    c. Staff pathologist **[THANK AND TERMINATE]**
    d. Laboratory tech, lead tech, or medical tech **[THANK AND TERMINATE]**

11. **[If (A) Selected in Q1]** Which sections of the laboratory do you manage and/or supervise? Select all that apply.
    a. Histology
    b. Cytology
    c. Cytogenetics
    d. Flow cytometry
    e. Clinical chemistry
    f. Immunoassay
    g. Coagulation
    h. Hematology
    i. Urinalysis
    j. Molecular (e.g., PCR, NGS, etc.) **[THANK AND TERMINATE IF NOT SELECTED]**
    k. Microbiology

12. **[If (A) Selected in Q1]** For which of the following clinical areas do you directly supervise/manage testing? Testing may be performed either in-house or as send-outs. Select all that apply.
    a. Virology (e.g., HIV, HCV testing)
    b. Women's health (e.g., HPV, STI testing)
    c. Infectious disease (e.g., MRSA, *C. difficile* testing)
    d. Oncology (e.g., BRAF mutation testing)
    e. Rare/Undiagnosed Diseases
    f. Pharmacogenomics
    g. Prenatal/Neonatal Screening
    h. Healthy Patient Screening

[THANK AND TERMINATE IF ONCOLOGY, RARE/UNDIAGNOSED DISEASES, PHARMACOGENOMICS, PRENATAL/NEONATAL SCREENING OR HEALTHY PATIENT SCREENING NOT SELECTED]

13. **[If (F) Selected in Q1]** Which of the following best describes your role?
    a. Administrator **[THANK AND TERMINATE]**
    b. Director **[THANK AND TERMINATE]**
    c. Manager **[THANK AND TERMINATE]**
    d. Supervisor **[THANK AND TERMINATE]**
    e. Vice President
    f. C-Suite Executive
    g. Other **[THANK AND TERMINATE]**

14. **[If (C) Selected in Q1 OR (F) Selected in Q13]** Which of the following best describes your title?
    a. Chief Informatics Officer
    b. Chief Medical Informatics Officer/Chief Clinical Informatics Officer
    c. Chief Medical Officer
    d. Chief Nursing Informatics Officer **[THANK AND TERMINATE]**
    e. Chief Executive Officer **[THANK AND TERMINATE]**
    f. Chief Financial Officer **[THANK AND TERMINATE]**
    g. Other **[THANK AND TERMINATE]**

15. **[If (A) Selected in Q14]** Which of the following degrees do you hold? Select all that apply.
    a. MD
    b. PhD
    c. MBA
    d. Other Master's
    e. Bachelor's
    f. Other (please specify)

[THANK AND TERMINATE IF (A) SELECTED IN Q14 AND (A) NOT SELECTED IN Q15]

16. **[If (C) or (F) Selected in Q1]** What responsibilities fall under your purview? Select all that apply.
    a. Selecting enterprise healthcare technology for health system
    b. Ensuring reliability, scalability, and affordability of IT infrastructure
    c. Optimizing quality, safety, and efficiency in clinician workflows
    d. Establishing clinician consensus for decision support rules
    e. Supporting regulatory compliance and privacy protections
    f. Integrating genomic data into medical records
    g. Implementing telehealth solutions

[THANK AND TERMINATE IF (F) NOT SELECTED]

17. **[If (C) or (F) Selected in Q1]** Which of the following best describes your involvement in technology purchasing decisions?
    a. I am the sole decision-maker
    b. I make the decision jointly with my colleagues
    c. I am a member of a committee that makes the decision
    d. I understand how the decision is made but am not part of the process **[THANK AND TERMINATE]**
    e. I am not part of nor have visibility into the decision-making process **[THANK AND TERMINATE]**

18. Which of the following best describes the integration of genomic data into your organization's EHR?
    a. Genomic data is scanned or uploaded in PDF form to a patient's EHR record
    b. The EHR has specific structured data fields for genomic data; these fields must be manually populated

c. The EHR has specific structured data fields for genomic data; these fields are automatically populated
d. I don’t know **[THANK AND TERMINATE]**

19. Which of the following best describes your involvement in your organization’s personalized medicine initiatives?
    a. I spearhead personalized medicine initiatives
    b. I am a member of a committee that oversees personalized medicine initiatives
    c. I am well aware of my organization’s personalized medicine initiatives across therapeutic areas, but am not directly involved
    d. I am only somewhat aware of my organization’s personalized medicine initiatives and am not directly involved **[THANK AND TERMINATE]**
    e. I am neither aware of nor involved in my organization’s personalized medicine initiatives **[THANK AND TERMINATE]**
